# Supplementary material for: Nox4 regulates InsP3 receptor‐dependent Ca2+ release into mitochondria to promote cell survival
Source: EMBO J. 2020 Aug 10;39(19):e103530. doi: 10.15252/embj.2019103530 (PMC7527947; doi:10.15252/embj.2019103530)
Supplement: Supplementary file 1 — Appendix [file EMBJ-39-e103530-s001.pdf]

APPENDIX

Beretta et al., Nox4 regulates $InsP_3$ receptor Ca^{2+} release to mitochondria to promote cell survival.

Table of Contents

Appendix Figure S1

Appendix Figure S2

Appendix Figure S3

Appendix Figure S4

Appendix Figure S5

Appendix Figure S6

Appendix Figure S1. Regulated cell necrosis is inhibited by Nox4.

A Nox4 mRNA levels in cultured rat cardiomyocytes after serum starvation for different durations, as compared to serum-replete cells. n=4/group.

B, C Immunoblotting for cleaved caspase-3 (Casp3, ***C***) or cleaved caspase-8 (Casp8, ***D***) in serum-starved cardiomyocytes depleted of Nox4 (Ad.shNox4) versus control (Ad.Ctl). Incubation with staurosporine (Stauro,1 μmol/L) was used as a pro-apoptotic control. Representative blots are shown in the inset panels. n=4/group.

D Immunoblot analyses in serum-starved cultured cardiomyocytes for levels of proteins involved in different pathways of regulated cell death (RCD). Results are shown as percentage change after serum starvation relative to baseline (dotted line). RIPK1/3 = receptor-interacting protein kinases 1/3; AIF = apoptosis-inducing factor; tAIF = truncated AIF; PAR = poly-ADP-ribosylation of proteins. n=5-8/group.

E Effect of bongkrekic acid (BA) on cell death in serum-starved cardiomyocytes. n=3/group.

F Cytoplasmic cytochrome *c* protein levels as a readout for apoptosis in serum-starved WT and Nox4KO MEFs. Some WT MEFs were treated with PEG-catalase (Cat, 500 U/mL). Nox4KO MEFs were transfected with active Nox4 or catalytically inactive mutant, $Nox4^{P437H}$ (Mut). A representative blot is shown at the top. The membrane fraction (Mem) containing mitochondria was used as a positive control. n=4/group.

G Transfection of Nox4KO MEFs with active Nox4 or a catalytically inactive mutant (Mut). The immunoblot shows levels of expressed protein. The graph at the bottom shows levels of H_2O_2 in the medium measured using an Amplex red assay. Ctl, untransfected Nox4KO MEFs. n=4/group.

Data are mean ± SEM. * $P<0.05$; ** $P<0.01$; *** $P<0.001$; **** $P<0.0001$ among compared groups or vs control. A: 2-way ANOVA; B,C,E-G: 1-way ANOVA with Tukey's post-test; D: unpaired t-test.

Appendix Figure S2. Nox4 and mitochondrial calcium levels.

A Basal mitochondrial calcium levels measured using a mitochondrial-targeted CFP/YFP FRET probe (Cameleon) in serum-starved cultured rat cardiomyocytes after siRNA-mediated knockdown of Nox4 or treatment with a scrambled siRNA (siScr). Top panels show YFP and bottom panels CFP fluorescence. After subtraction of the background signal, images were thresholded and the YFP/CFP ratio was calculated to obtain the images shown in ***Fig 2A***. Scale bars: 10 µm.

B Basal mitochondrial calcium levels in serum-starved WT and Nox4KO MEFs, as in (***A)***. Corresponds to the images shown in ***Fig 2B***. Scale bars: 10 µm.

C, D Changes in cytosolic calcium levels in serum-starved WT and Nox4KO MEFs after the addition of histamine (100 µmol/L, ***C***) or ATP (100 µmol/L, ***D***). n=3/group (with >30 cells per individual experiment).

E Time course of changes in mitochondrial calcium levels in serum-replete WT and Nox4KO MEFs after treatment with histamine. n=3/group (with >30 cells imaged per experiment). Representative images of the YFP/CFP ratio are shown to the bottom. Scale bars: 10 µm. 2-way repeated measures ANOVA.

Appendix Figure S3. Nox4 localization at the MAM by electron microscopy.

A, B Proportion of Nox4 or FACL4 immunolabeling observed at the MAM relative to overall labeling in sections from WT and Nox4 KO hearts (***A***) or hiPSC-CM with or without Nox4 knockdown (Nox4 KD). >25 micrographs per condition were analyzed.

Data are mean ±SEM. **** P<0.0001 vs corresponding control (unpaired t-test).

Appendix Figure S4. Nox4 is in proximity of FACL4 and InsP$_3$R.

A Confocal photomicrographs of co-localization studies in WT MEFs. Cells were probed with Nox4 antibody (green) and either FACL4 or InsP$_3$R antibody (red). Scale bars: 10 µm.

B Simplified photomicrographs of proximity ligation studies in WT and Nox4KO MEFs. Quantification of the number of dots/cell in each condition is shown to the right. Proximity was tested for the following proteins: control staining with a single Ab for FACL4, $InsP_3R$, Nox4 or LAMP1; the protein couple LAMP1/Nox4. LAMP1, lysosomal associated membrane protein 1. n=3/group (with >30 cells/individual experiment). Original unsimplified pictures are shown in ***Appendix Fig S4E***. Scale bars: 10 µm.

C Simplified photomicrographs of proximity ligation studies in rat cardiomyocytes transfected with Nox4 siRNA or a scrambled control, with quantification as in (***B)***. Proximity was tested for the following proteins: control staining with a single Ab for FACL4, $InsP_3R$ or Nox4; the protein couples $InsP_3R$/Nox4 and FACL4/$InsP_3R$. n=3/group (with >30 cells/individual experiment). Original unsimplified pictures are shown in ***Appendix Fig S4F***. Scale bars: 10 µm.

D Simplified photomicrographs of proximity ligation staining in hiPSC-CM (human cardiomyocytes) transduced with Nox4 shRNA or scrambled control), with quantification as in (***B)***. Proximity was tested for the following proteins: control staining with a single Ab for FACL4, $InsP_3R$ or Nox4; the protein couples $InsP_3R$/Nox4 and FACL4/$InsP_3R$. n=3/group (with >30 cells/individual experiment). Original unsimplified pictures are shown in ***Appendix Fig S4G.*** Scale bars: 10 µm.

E Original photomicrographs taken for proximity ligation assay (PLA) in WT and Nox4KO MEFs. Control staining with single antibodies (FACL4, $InsP_3R$, Nox4, LAMP1). PLA was made for the combinations FACL4/Nox4, $InsP_3R$/Nox4, FACL4/$InsP_3R$, and LAMP1/Nox4. Blue, DAPI staining; Green, PLA staining (dots); Red, actin staining. Scale bars: 10 µm. Simplified pictures are shown in ***Figs 4A and Appendix Fig S4B***.

F Original photomicrographs taken for PLA in rat cardiomyocytes transfected with Nox4 siRNA or a scrambled control. Control staining with single antibodies (FACL4, $InsP_3R$, Nox4). PLA was made for the combinations FACL4/Nox4, $InsP_3R$/Nox4, and FACL4/$InsP_3R$. Blue, DAPI staining; Green, PLA staining (dots); Red, actin staining. Scale bars: 10 µm. Simplified pictures are shown in ***Figs 4B and Appendix Fig S4C***.

G Original photomicrographs taken for PLA in hiPSC-CM transduced with Nox4 shRNA or scrambled control. Control staining with single antibodies (FACL4, $InsP_3R$, Nox4). PLA was made for the combinations FACL4/Nox4, $InsP_3R$/Nox4, and FACL4/$InsP_3R$. Blue, DAPI staining; Green, PLA staining (dots); Red, actin staining. Scale bars, 10 µm. Simplified pictures are shown in ***Figs 4C and Appendix Fig S4D***.

H Simplified photomicrographs of proximity ligation studies in Nox4KO MEFs after transfection with active Nox4 or rat cardiomyocytes transfected with Nox4 siRNA or a $Nox4^{P437H}$ mutant (Mut). Proximity was tested for the following protein couples: FACL4/Nox4, $InsP_3R$/Nox4 and FACL4/$InsP_3R$. n=3/group (with >30 cells/individual experiment). Quantification as in (***B)***. Scale bars: 10 µm.

Data are mean ± SEM. **** P<0.0001 vs corresponding control (unpaired t-test).

Appendix Figure S5. Regulation of Akt activation and $InsP_3R$ phosphorylation by Nox4.

A Approach used to detect Akt-mediated phosphorylation of $InsP_3R$ phosphorylation. After immunoprecipitation (IP) of $InsP_3R$, the sample was immunoblotted (IB) both for $InsP_3R$ and the phosphorylated Akt substrate motif (RXRXX-pS/T). IP with IgG was used as a control.

B Immunoblotting for phosphorylated and total Akt and $InsP_3R$ in crude mitochondrial fractions isolated from serum-replete WT and Nox4KO MEFs. $InsP_3R$ and p-$InsP_3R$ were detected after IP for $InsP_3R$ as described in A. n=3/group.

C ROS levels indexed in serum-replete WT and Nox4 KO MEFs using H_2O_2 specific HyPer probes targeted to the MAM (FACL4 HyPer) or the cytosol (Cyto HyPer). Imaging and analysis was performed as in ***Fig 5D***. Representative photomicrographs of the fluorescence ratio are shown to the left and mean data for changes in fluorescence ratio to the right. n=5 independent cell preparations/group, with at least 20 cells imaged/preparation. Scale bars: 10 µm. * P<0.05, ** P<0.001 compared to WT control group. # P<0.05 vs Nox4KO group (1-way ANOVA with Tukey's post-test).

D ROS-insensitive SypHer-probes were used as controls for FACL4-HyPer and cytosolic HyPer probes, to correct for any pH-dependent changes in fluorescence. The images show the responses of the 4 probes to addition of H_2O_2 (200 µmol/L). Scale bars: 10 µm.

Data are mean ± SEM.

Appendix Figure S6. Functional effects of Nox4-dependent regulation of $InsP_3R$.

A Effect of treatment with okadaic acid (OA) on $InsP_3R$ and Akt phosphorylation in serum-starved WT and Nox4KO MEFs. Mean data shown to the right. n=3/group.

B Immunoblotting to quantify the phosphorylation levels of Akt and $InsP_3R$ in crude mitochondrial fractions prepared from WT and Nox4KO hearts at baseline. Mean data shown to the right. n=3/group.

Data are mean ± SEM. *** P<0.001 compared to WT control group. ## P<0.01, ### P<0.001 vs Nox4KO group (1-way ANOVA with Tukey's post-test).

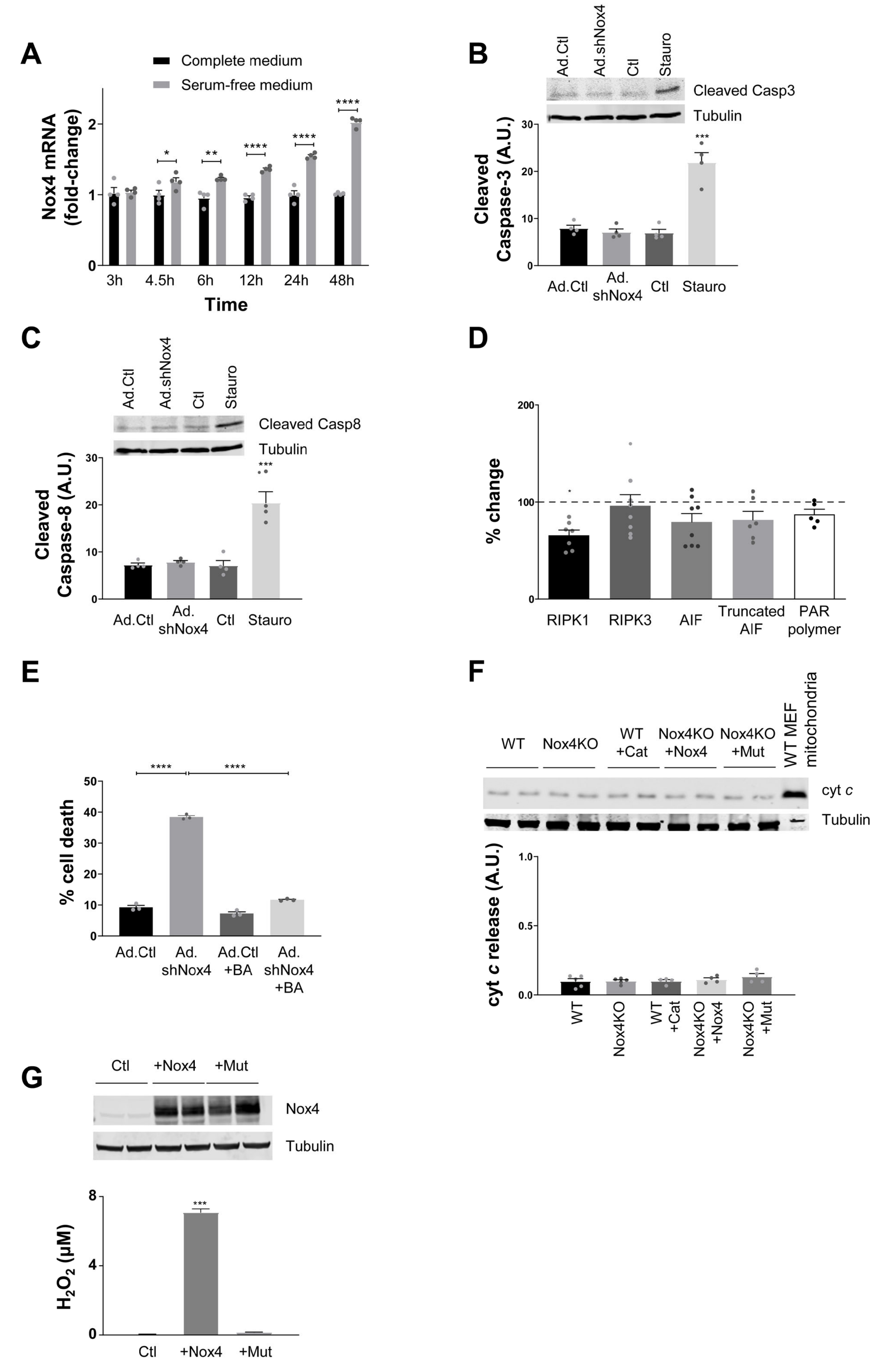
A
Complete medium
Serum-free medium
Nox4 mRNA (fold-change)
3h
4.5h
6h
12h
24h
48h
Time
B
Ad.Ctl
Ad.shNox4
Ctl
Stauro
Cleaved Casp3
Tubulin
Cleaved Caspase-3 (A.U.)
C
Cleaved Casp8
Cleaved Caspase-8 (A.U.)
D
% change
RIPK1
RIPK3
AIF
Truncated AIF
PAR polymer
E
% cell death
Ad.Ctl +BA
Ad. shNox4 +BA
F
WT
Nox4KO
WT +Cat
Nox4KO +Nox4
Nox4KO +Mut
WT MEF mitochondria
cyt c
cyt c release (A.U.)
G
Ctl
+Nox4
+Mut
Nox4
H_2O_2 (μM)

Figure S1

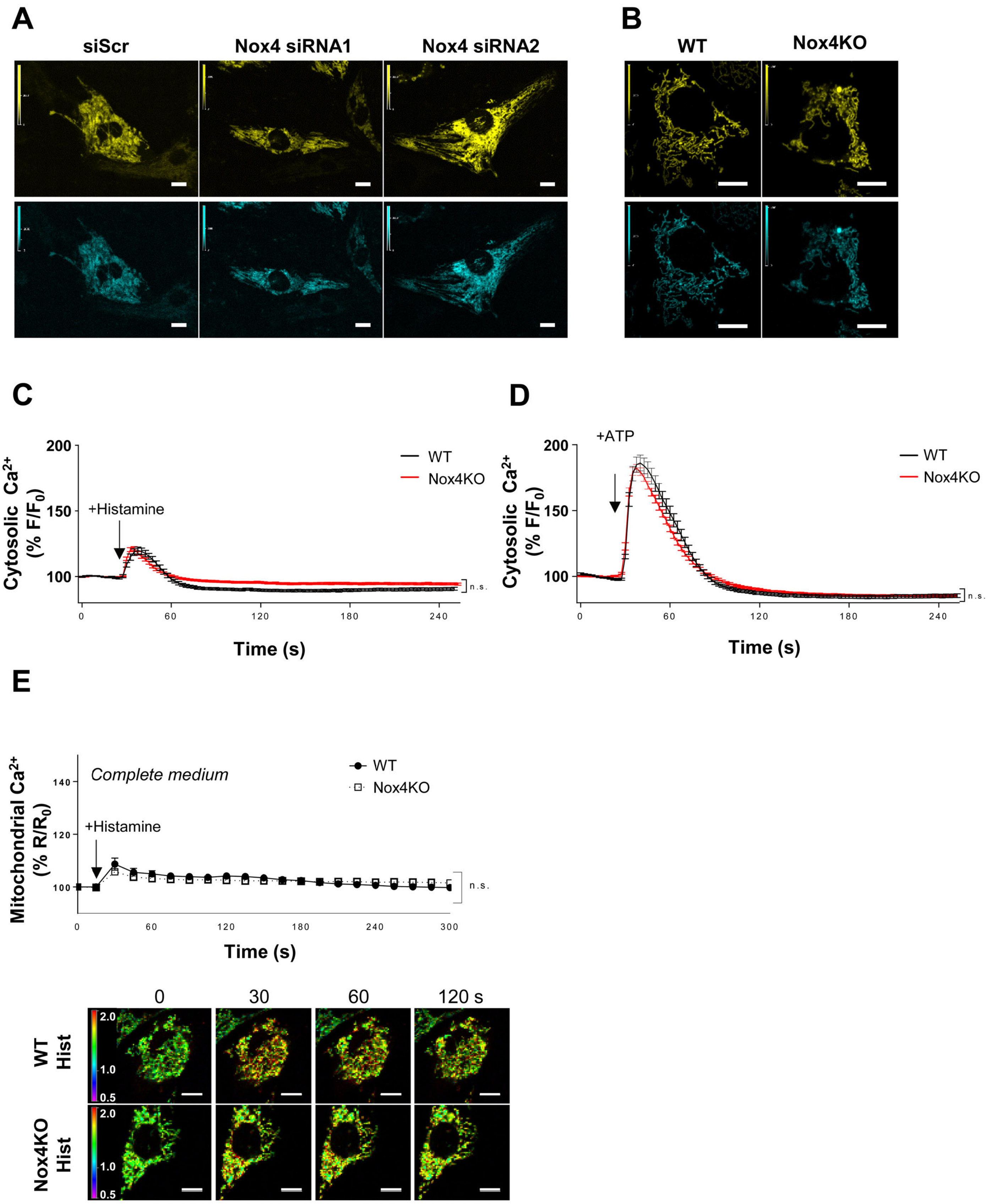
A
siScr
Nox4 siRNA1
Nox4 siRNA2
B
WT
Nox4KO
C
Cytosolic Ca2+ (% F/F0)
+Histamine
WT
Nox4KO
n.s.
Time (s)
D
+ATP
Cytosolic Ca2+ (% F/F0)
WT
Nox4KO
n.s.
Time (s)
E
Mitochondrial Ca2+ (% R/R0)
Complete medium
+Histamine
WT
Nox4KO
n.s.
Time (s)
0
30
60
120 s
WT Hist
Nox4KO Hist
2.0
1.0
0.5

Figure S2

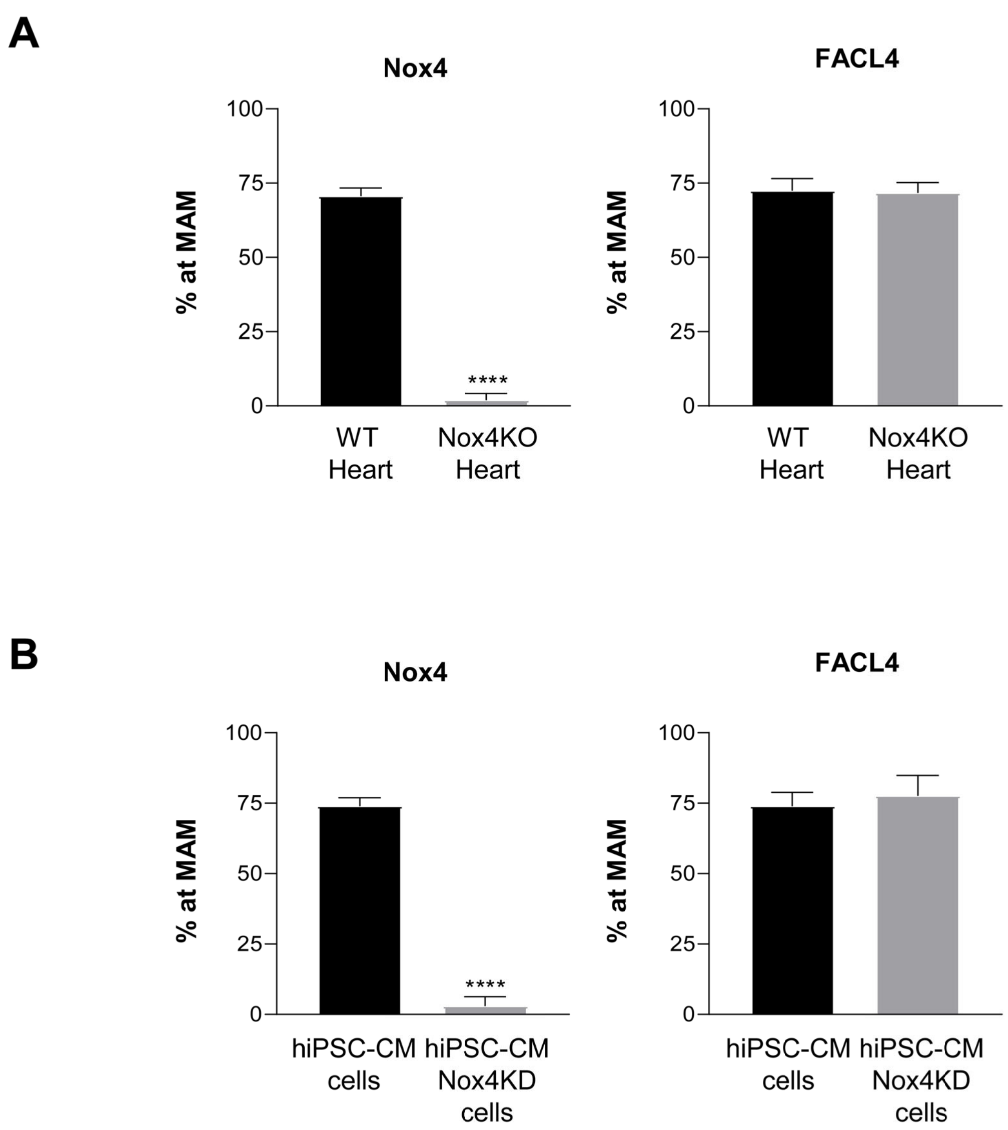
A
Nox4
FACL4
% at MAM
100
75
50
25
0
****
WT Heart
Nox4KO Heart
B
Nox4
FACL4
% at MAM
hiPSC-CM cells
hiPSC-CM Nox4KD cells

Figure S3

A

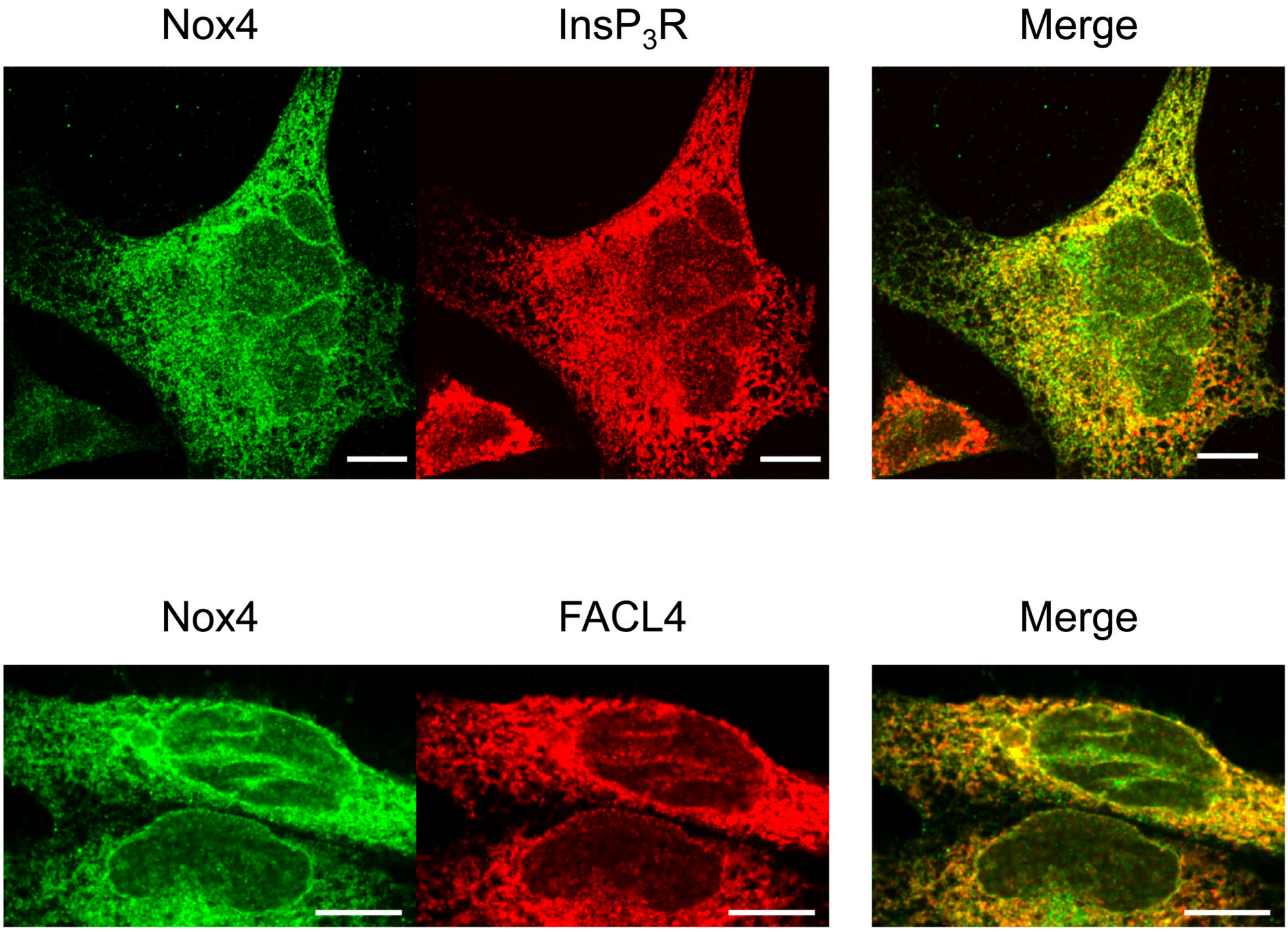

Figure S4

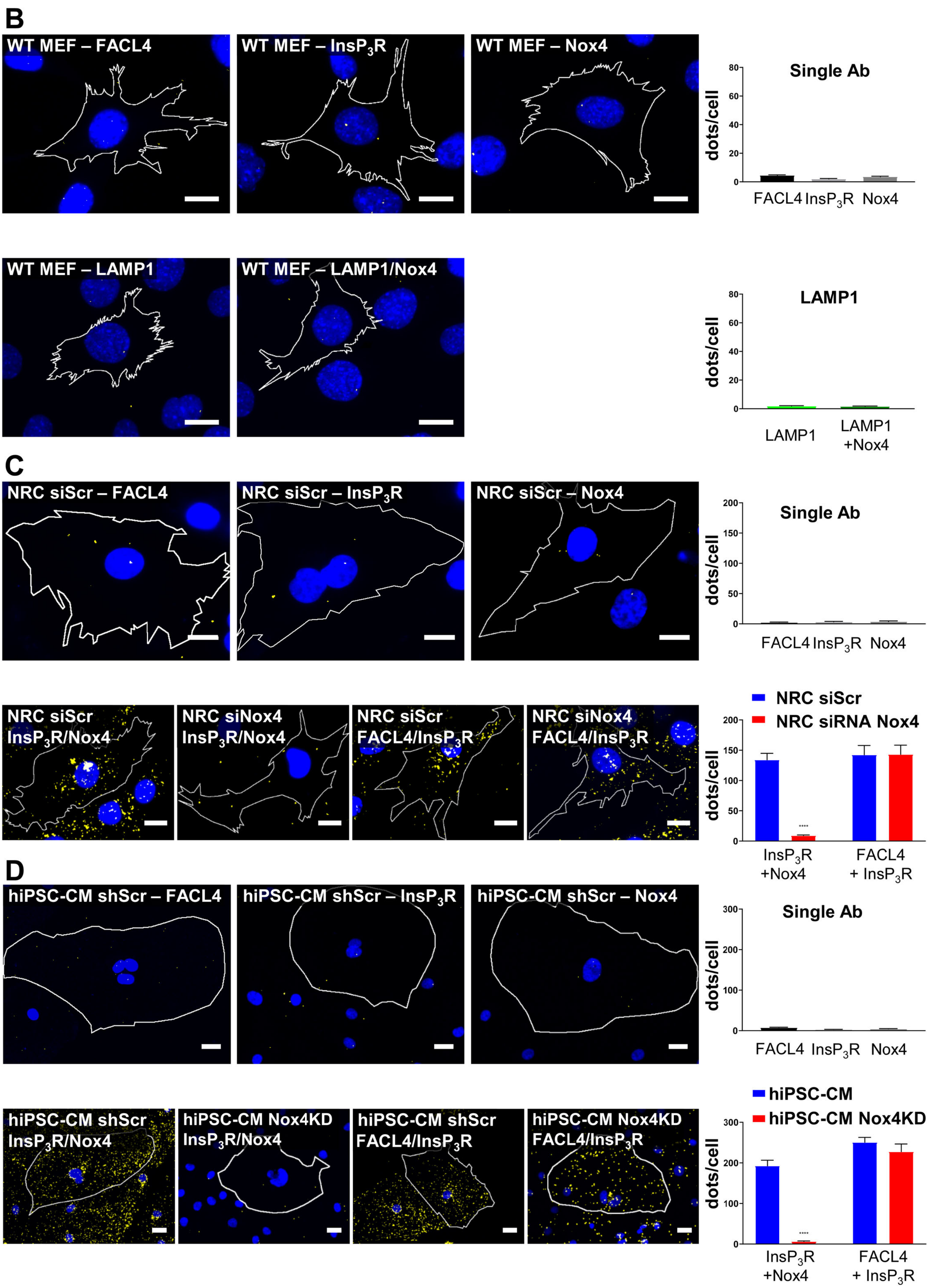
B
WT MEF – FACL4
WT MEF – InsP3R
WT MEF – Nox4
Single Ab
dots/cell
FACL4 InsP3R Nox4
WT MEF – LAMP1
WT MEF – LAMP1/Nox4
LAMP1
LAMP1
LAMP1 +Nox4
C
NRC siScr – FACL4
NRC siScr – InsP3R
NRC siScr – Nox4
Single Ab
NRC siScr InsP3R/Nox4
NRC siNox4 InsP3R/Nox4
NRC siScr FACL4/InsP3R
NRC siNox4 FACL4/InsP3R
NRC siScr
NRC siRNA Nox4
InsP3R +Nox4
FACL4 + InsP3R
D
hiPSC-CM shScr – FACL4
hiPSC-CM shScr – InsP3R
hiPSC-CM shScr – Nox4
Single Ab
hiPSC-CM shScr InsP3R/Nox4
hiPSC-CM Nox4KD InsP3R/Nox4
hiPSC-CM shScr FACL4/InsP3R
hiPSC-CM Nox4KD FACL4/InsP3R
hiPSC-CM
hiPSC-CM Nox4KD

Figure S4

E

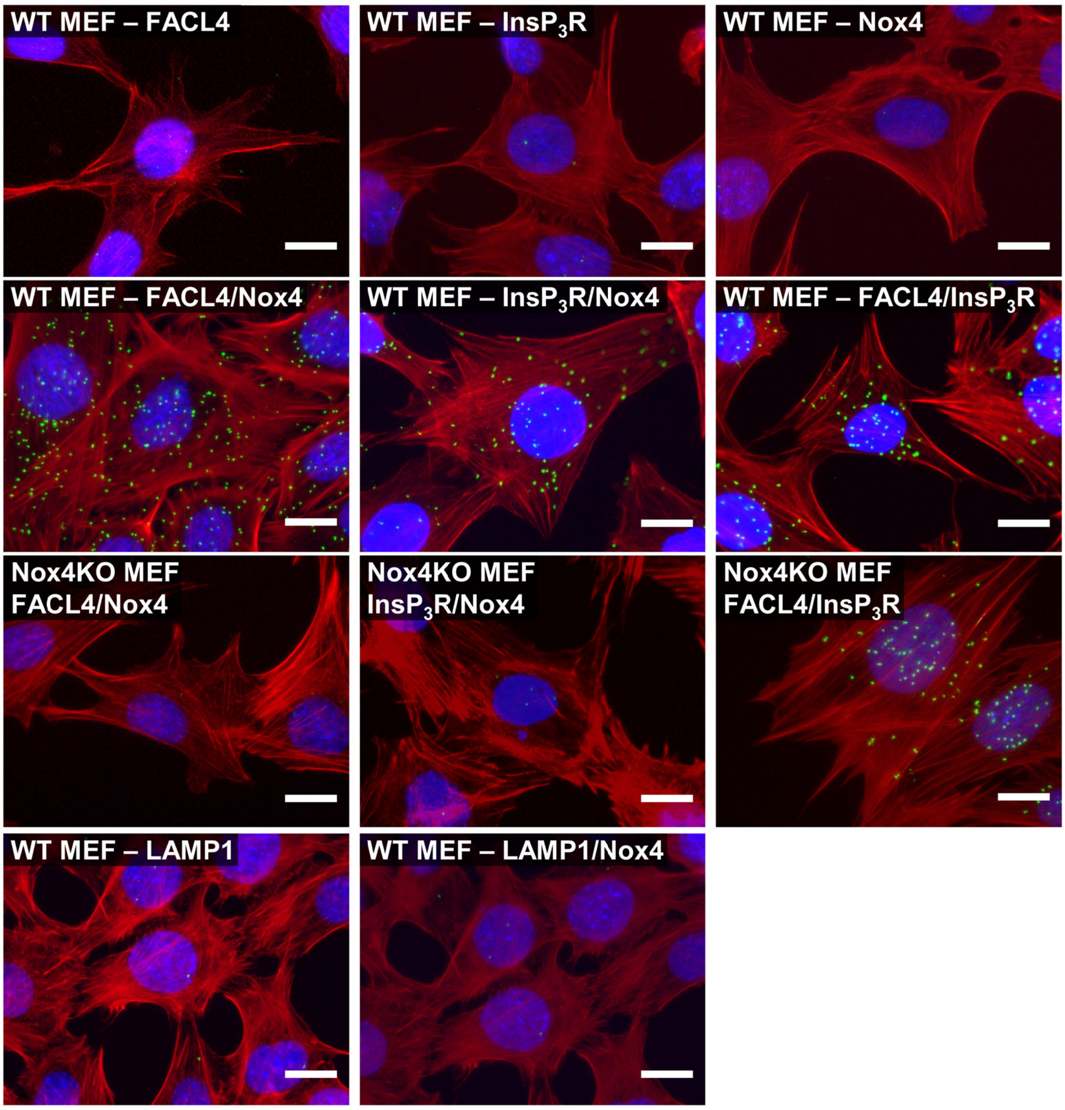
WT MEF – FACL4
WT MEF – InsP3R
WT MEF – Nox4
WT MEF – FACL4/Nox4
WT MEF – InsP3R/Nox4
WT MEF – FACL4/InsP3R
Nox4KO MEF FACL4/Nox4
Nox4KO MEF InsP3R/Nox4
Nox4KO MEF FACL4/InsP3R
WT MEF – LAMP1
WT MEF – LAMP1/Nox4

Figure S4

F

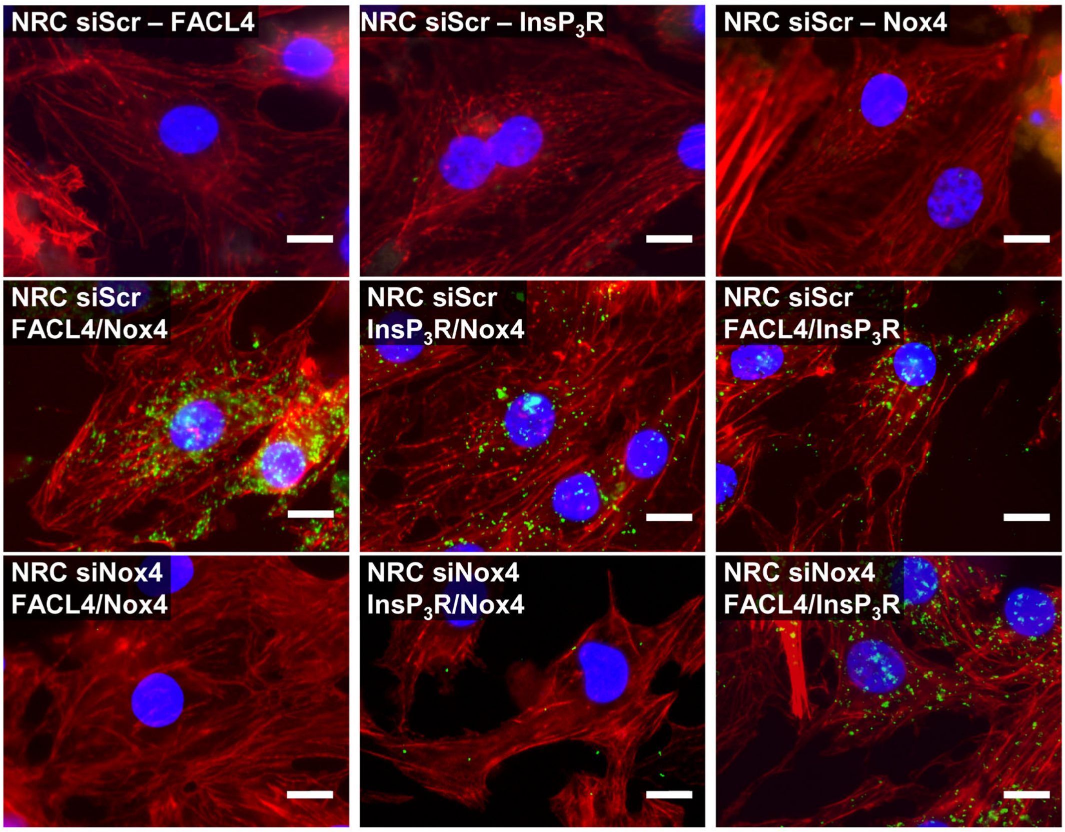
NRC siScr – FACL4
NRC siScr – InsP$_3$R
NRC siScr – Nox4
NRC siScr FACL4/Nox4
NRC siScr InsP$_3$R/Nox4
NRC siScr FACL4/InsP$_3$R
NRC siNox4 FACL4/Nox4
NRC siNox4 InsP$_3$R/Nox4
NRC siNox4 FACL4/InsP$_3$R

G

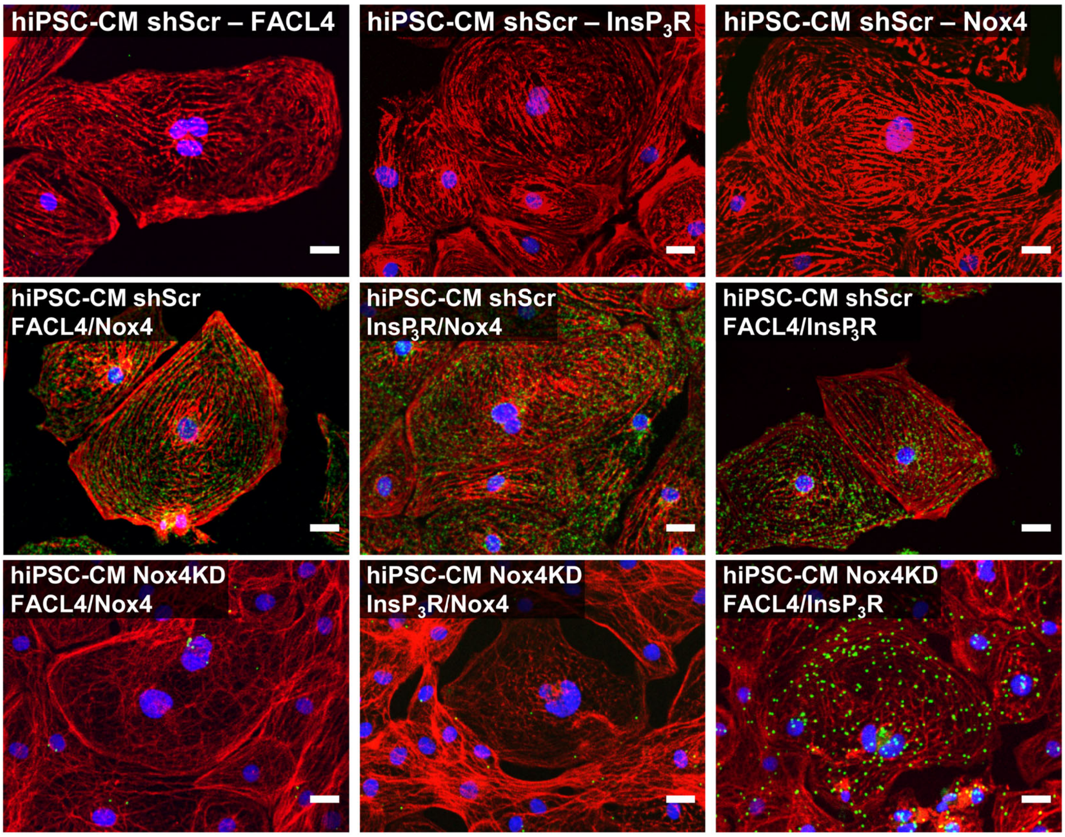
hiPSC-CM shScr – FACL4
hiPSC-CM shScr – InsP$_3$R
hiPSC-CM shScr – Nox4
hiPSC-CM shScr FACL4/Nox4
hiPSC-CM shScr InsP$_3$R/Nox4
hiPSC-CM shScr FACL4/InsP$_3$R
hiPSC-CM Nox4KD FACL4/Nox4
hiPSC-CM Nox4KD InsP$_3$R/Nox4
hiPSC-CM Nox4KD FACL4/InsP$_3$R

Figure S4

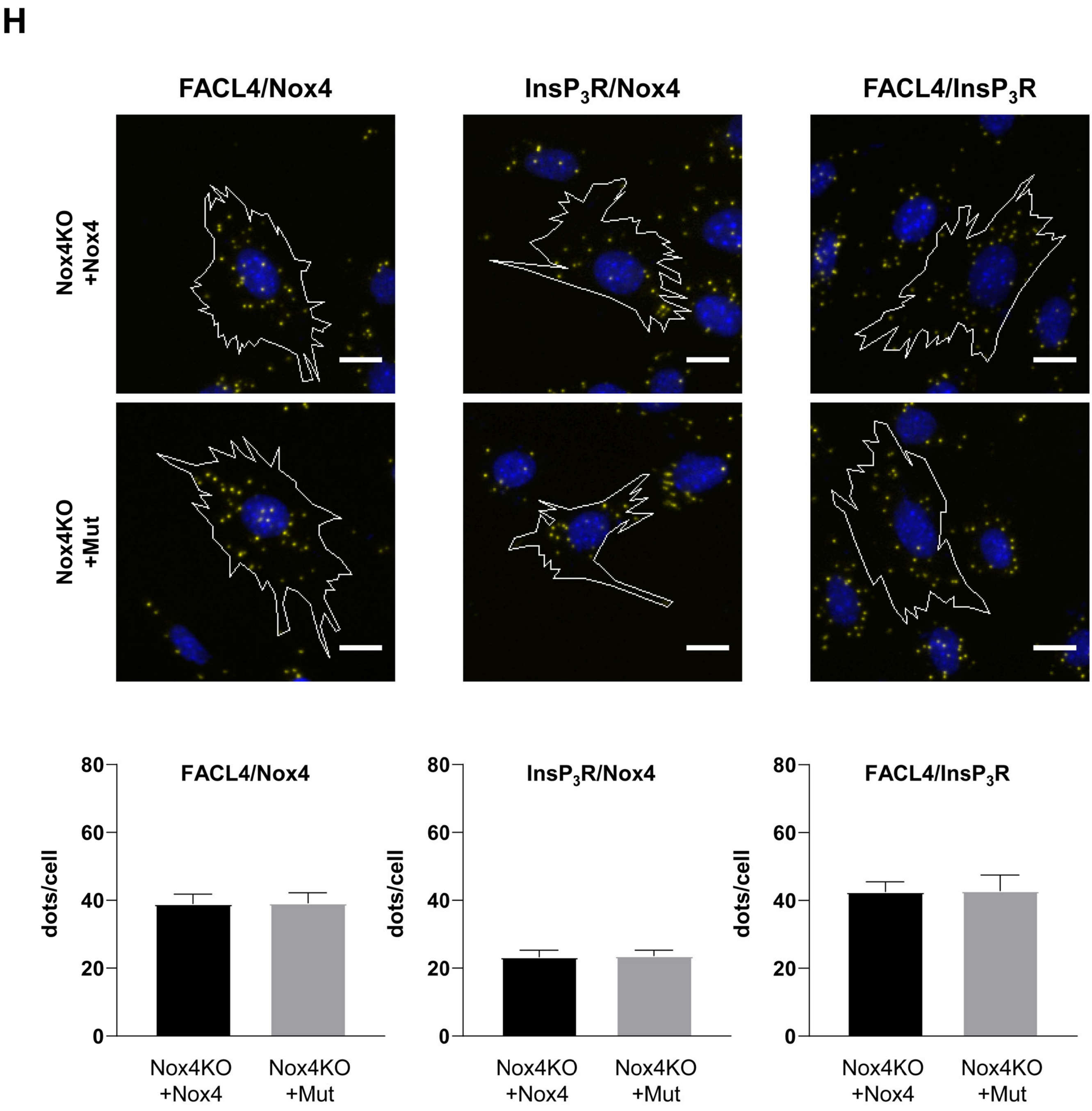
H
FACL4/Nox4
InsP3R/Nox4
FACL4/InsP3R
Nox4KO +Nox4
Nox4KO +Mut
dots/cell
80
60
40
20
0
Nox4KO +Nox4
Nox4KO +Mut

Figure S4

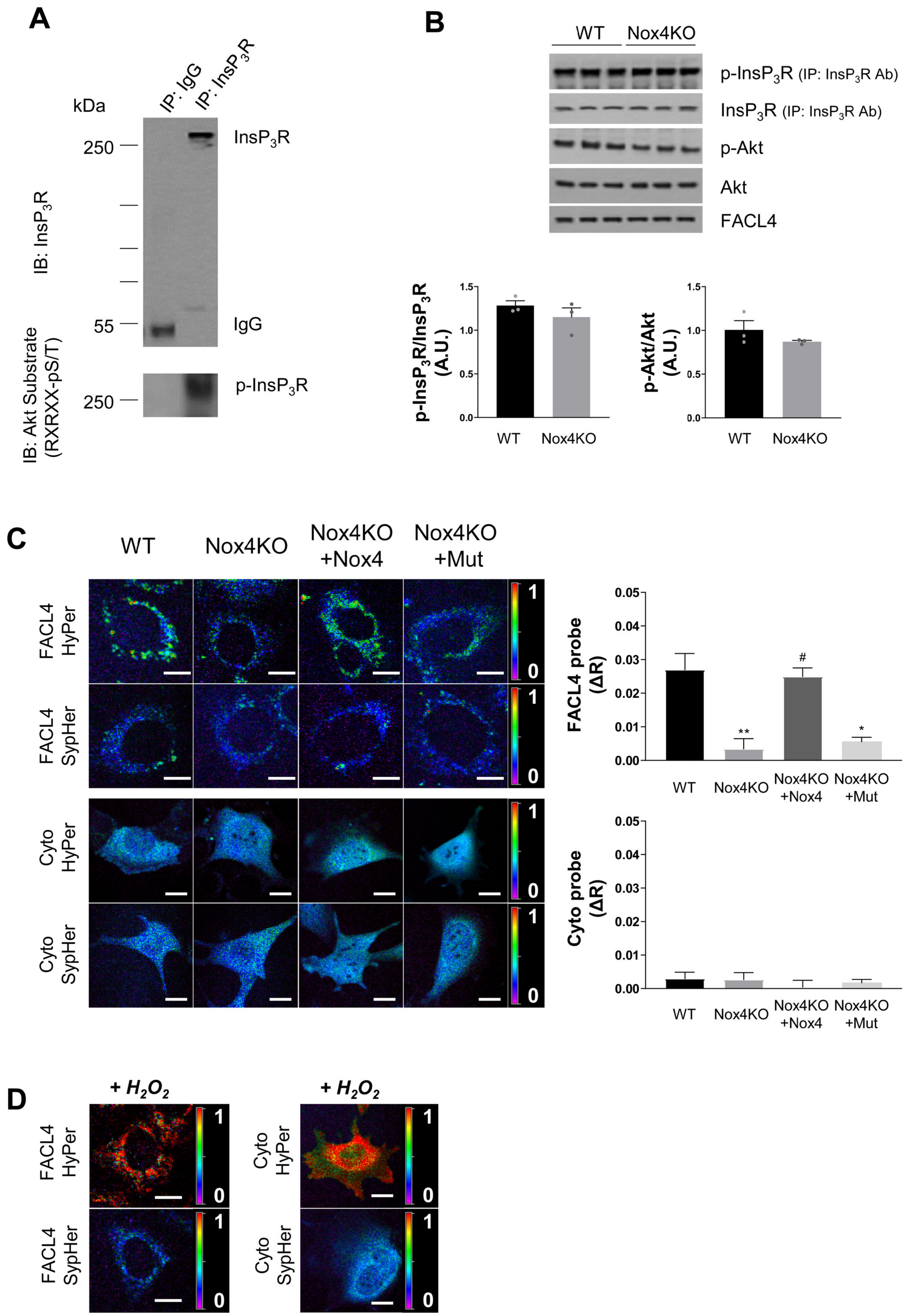
A
kDa
IP: IgG
IP: InsP3R
250
55
250
InsP3R
IgG
p-InsP3R
IB: InsP3R
IB: Akt Substrate (RXRXX-pS/T)
B
WT
Nox4KO
p-InsP3R (IP: InsP3R Ab)
InsP3R (IP: InsP3R Ab)
p-Akt
Akt
FACL4
p-InsP3R/InsP3R (A.U.)
p-Akt/Akt (A.U.)
1.5
1.0
0.5
0.0
WT
Nox4KO
C
WT
Nox4KO
Nox4KO +Nox4
Nox4KO +Mut
FACL4 HyPer
FACL4 SypHer
Cyto HyPer
Cyto SypHer
1
0
FACL4 probe (ΔR)
Cyto probe (ΔR)
0.05
0.04
0.03
0.02
0.01
0.00
**
#
*
WT
Nox4KO
Nox4KO +Nox4
Nox4KO +Mut
D
+ H2O2
FACL4 HyPer
FACL4 SypHer
Cyto HyPer
Cyto SypHer

Figure S5

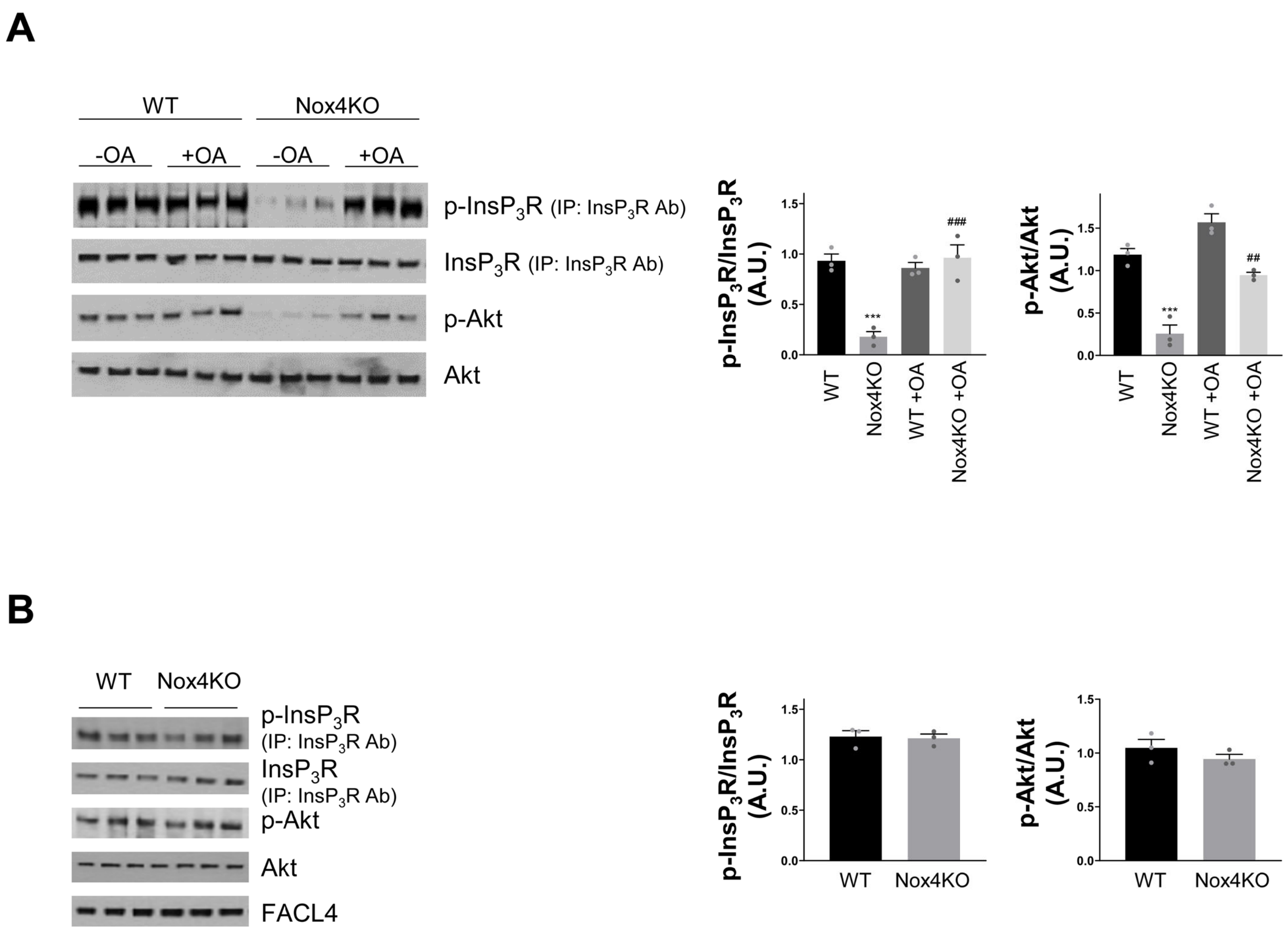
A
WT
Nox4KO
-OA
+OA
-OA
+OA
p-InsP3R (IP: InsP3R Ab)
InsP3R (IP: InsP3R Ab)
p-Akt
Akt
p-InsP3R/InsP3R (A.U.)
p-Akt/Akt (A.U.)
WT
Nox4KO
WT +OA
Nox4KO +OA
***
###
##
B
WT
Nox4KO
p-InsP3R (IP: InsP3R Ab)
InsP3R (IP: InsP3R Ab)
p-Akt
Akt
FACL4

Figure S6
