# Supplementary material for: Nox4 regulates InsP3 receptor‐dependent Ca2+ release into mitochondria to promote cell survival
Source: EMBO J. 2020 Aug 10;39(19):e103530. doi: 10.15252/embj.2019103530 (PMC7527947; doi:10.15252/embj.2019103530)
Supplement: Supplementary file 2 — Expanded View Figures PDF [file EMBJ-39-e103530-s002.pdf]

Expanded View Figures

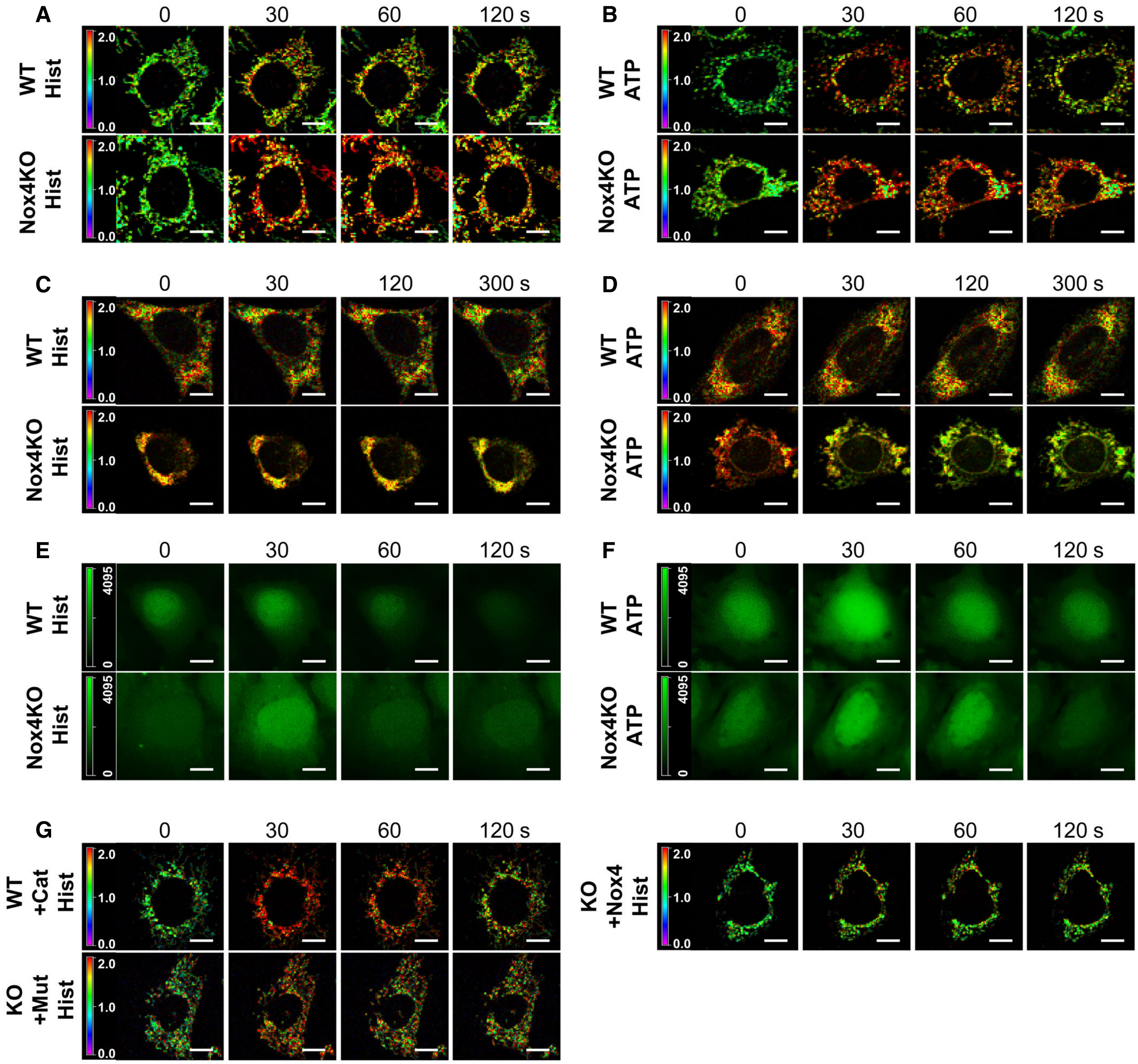

Figure EV1. Effect of Nox4 on subcellular calcium levels in stressed cells.

A, B Time course of changes in mitochondrial calcium levels in serum-starved WT and Nox4KO MEFs after treatment with histamine (Hist, A) or ATP (B). Images show the YFP/CFP ratio; agonists were added at t = 15 s. Related to Fig 2C and D, respectively. Scale bars: 10 µm.

C, D Time course of changes in ER calcium levels in serum-starved WT and Nox4KO MEFs after treatment with histamine (C) or ATP (D). Images show YFP/CFP ratio; agonists added at t = 15 s. Related to Fig 2E and F, respectively. Scale bars: 10 µm.

E, F Time course of changes in cytosolic calcium levels in serum-starved WT and Nox4KO MEFs after treatment with histamine (E) or ATP (F). Images show Fluo4 fluorescence. Related to Fig 2G and H, respectively. Scale bars: 10 µm.

G Time course of histamine-induced changes in mitochondrial calcium levels in serum-starved WT and Nox4KO MEFs as in (A). Cat = PEG-catalase. Nox4KO MEFs were transfected with active Nox4 or a catalytically inactive mutant (Mut). Relates to Fig 2I and J. Scale bars: 10 µm.

© 2020 The Authors

Figure EV2. Nox4 influences mitochondrial calcium levels via $InsP_3R$.

A, B Time course of histamine (Hist)-induced changes in mitochondrial calcium levels in serum-starved WT and Nox4 KO MEFs. $TMRE^+$ cells shown in (A) and $TMRE^-$ cells in (B). Sequential images to the right show YFP/CFP ratio, with quantification presented in the graphs. n = 3/group (with > 30 cells per individual experiment). Scale bars: 10 μm.

C Peak histamine-induced increase in mitochondrial calcium levels in serum-starved WT and Nox4 KO MEFs with or without treatment with bongkrekic acid (BA) to inhibit the mPTP. n = 3/group (with > 30 cells per individual experiment).

D Effect of caffeine or histamine on subcellular calcium levels in serum-starved cardiomyocytes depleted of Nox4 or treated with a control scrambled siRNA (siScr). Percent changes in peak calcium levels are shown. n = 3/group (with > 30 cells per individual experiment).

Data information: Data are mean ± SEM. $^{**}P < 0.01$; $^{***}P < 0.001$; $^{****}P < 0.0001$ among compared groups or vs. control. (A,B): 2-way repeated measures ANOVA; (C): 1-way ANOVA; (D): unpaired t-test.

 © 2020 The Authors

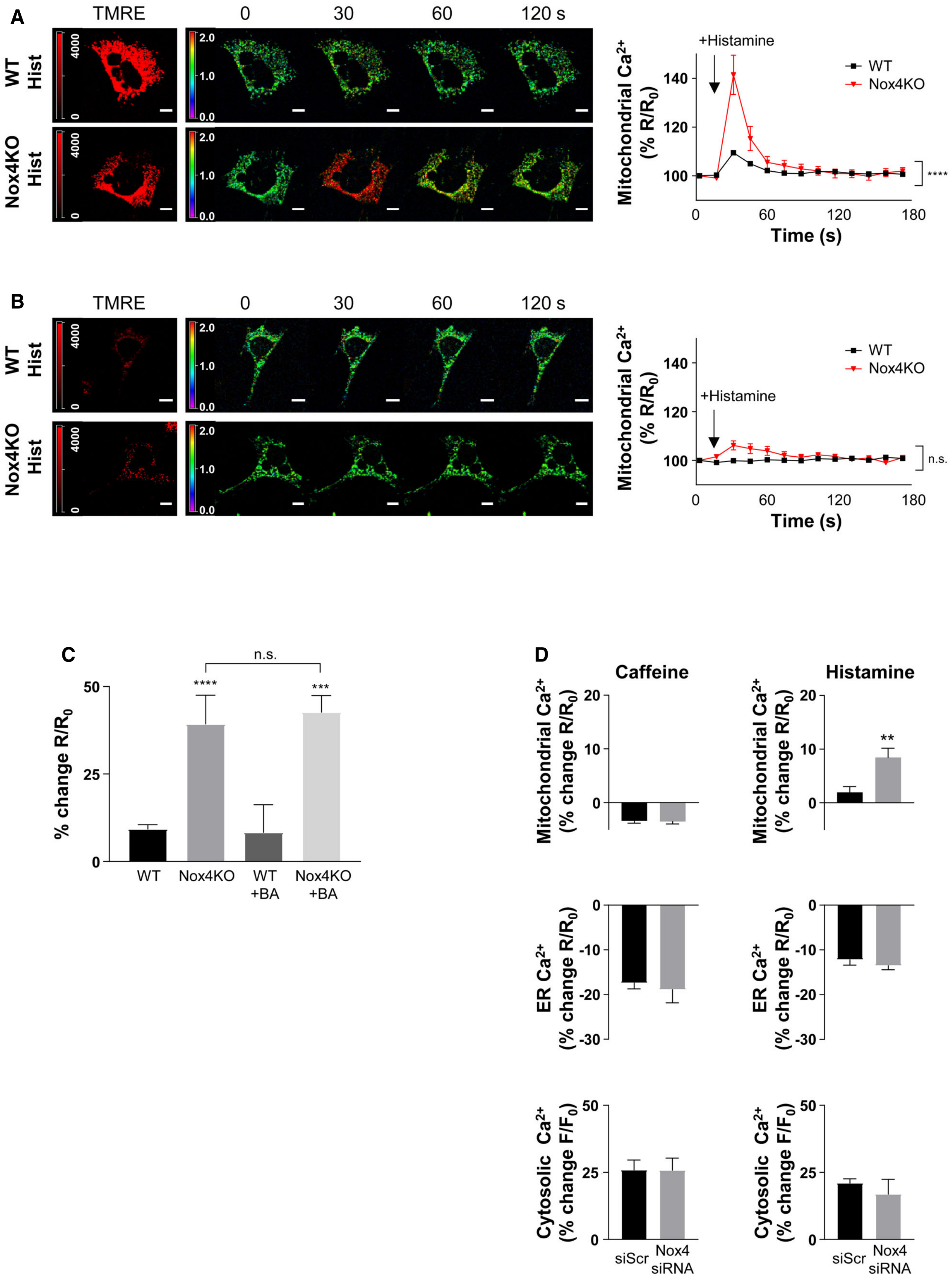
A
TMRE
0
30
60
120 s
WT Hist
Nox4KO Hist
+Histamine
WT
Nox4KO
Mitochondrial Ca2+ (% R/R0)
Time (s)
****
B
n.s.
C
n.s.
****
***
% change R/R0
WT
Nox4KO
WT +BA
Nox4KO +BA
D
Caffeine
Histamine
Mitochondrial Ca2+ (% change R/R0)
**
ER Ca2+ (% change R/R0)
Cytosolic Ca2+ (% change F/F0)
siScr
Nox4 siRNA

Figure EV2.

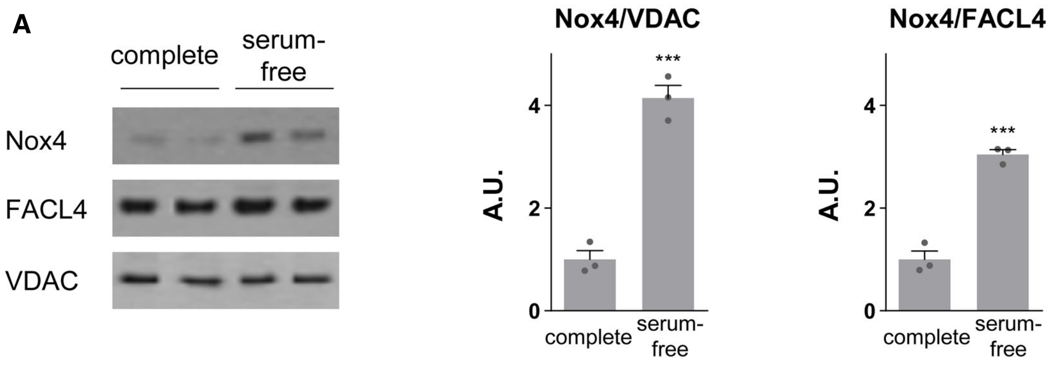

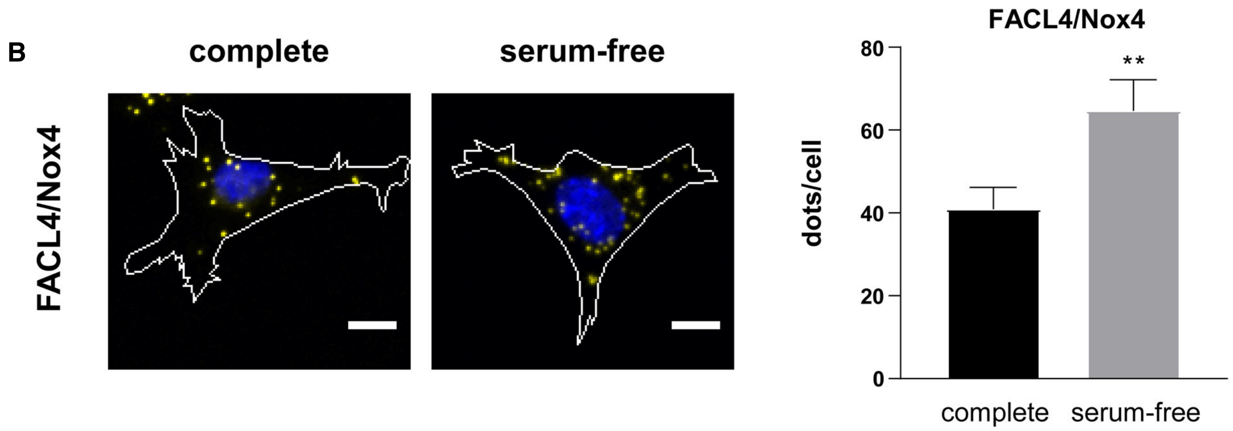

Figure EV3. Effect of serum starvation on Nox4 levels at the MAM.

A Effect of serum starvation on Nox4 protein levels in WT MEFs. FACL4 and VDAC used as MAM markers. n = 3/group.

B Simplified photomicrographs of proximity ligation studies in WT MEFs under serum-replete and serum-starved conditions (as in Fig 4). Nuclei are in blue, while yellow dots denote co-localization of proteins. Quantification of the number of dots/cell is shown to the right. Proximity was tested for FACL4/Nox4. Scale bars: 10 µm. n = 3/group (with > 50 cells/individual experiment).

Data information: Data are mean ± SEM. $^{**}P < 0.01$; $^{***}P < 0.001$ compared to control. Unpaired t-test.
Source data are available online for this figure.

 © 2020 The Authors

Figure EV4. Nox4 regulates serine/threonine phosphatase activity.

A Mitochondrial ROS levels in serum-starved WT and Nox4 KO MEFs indexed using a mitochondrial-targeted HyPer probe (Mito HyPer). Representative photomicrographs of the fluorescence ratio are shown to the left and mean data for changes in fluorescence ratio to the right. The corresponding ROS-insensitive SypHer probe was used to correct for any pH-induced changes in fluorescence. n = 3 independent cell preparations/group, with at least 50 cells imaged/preparation. Scale bars: 10 μm.

B Effect of Mito-TEMPO on the phosphorylation of $InsP_3R$ and Akt in serum-starved WT MEFs. $InsP_3R$ was first immunoprecipitated and then the precipitate was immunoblotted for total $InsP_3R$ and for the phosphorylated Akt substrate motif RXRXX(pS/T) (p-$InsP_3R$). FACL4 was used as a loading control for the MAM. Mean data shown to the bottom. n = 4/group.

C Effect of Mito-TEMPO on histamine-induced changes in mitochondrial calcium in serum-starved cells. Representative images of YFP/CFP ratio are shown to the right. n = 3/group (with > 30 cells per individual experiment). Scale bars: 10 μm.

D Ser/Thr phosphatase activity measured in crude mitochondrial fractions of MEFs in the presence or absence of okadaic acid (OA, 10 nmol/l; specific for PP2a inhibition) or calyculin A (Caly, 60 nmol/l; which inhibits PP1 and PP2a) or H_2O_2 (500 μmol/l). PP2a activity was calculated as the difference between control and OA, and PP1+PP2a activity as the difference between control and Caly. n = 5/group. $^{****}P < 0.0001$ compared to respective control; $^{\#}P < 0.05$, $^{\#\#\#}P < 0.001$, $^{\#\#\#\#}P < 0.0001$ comparing Nox4KO to respective WT group.

E Effect of treatment of cells with PEG-catalase (Cat) or transfection of Nox4KO cells with active Nox4 or mutant $Nox4^{P437H}$ (Mut) on Ser/Thr phosphatase activity. n = 4/group. $^{***}P < 0.001$, $^{****}P < 0.0001$ compared to WT control group; $^{\#\#\#\#}P < 0.0001$ compared to Nox4KO group.

Data information: Data are mean ± SEM. (A,B): unpaired t-test; (C): 2-way repeated measures ANOVA; (D, E): 1-way ANOVA.

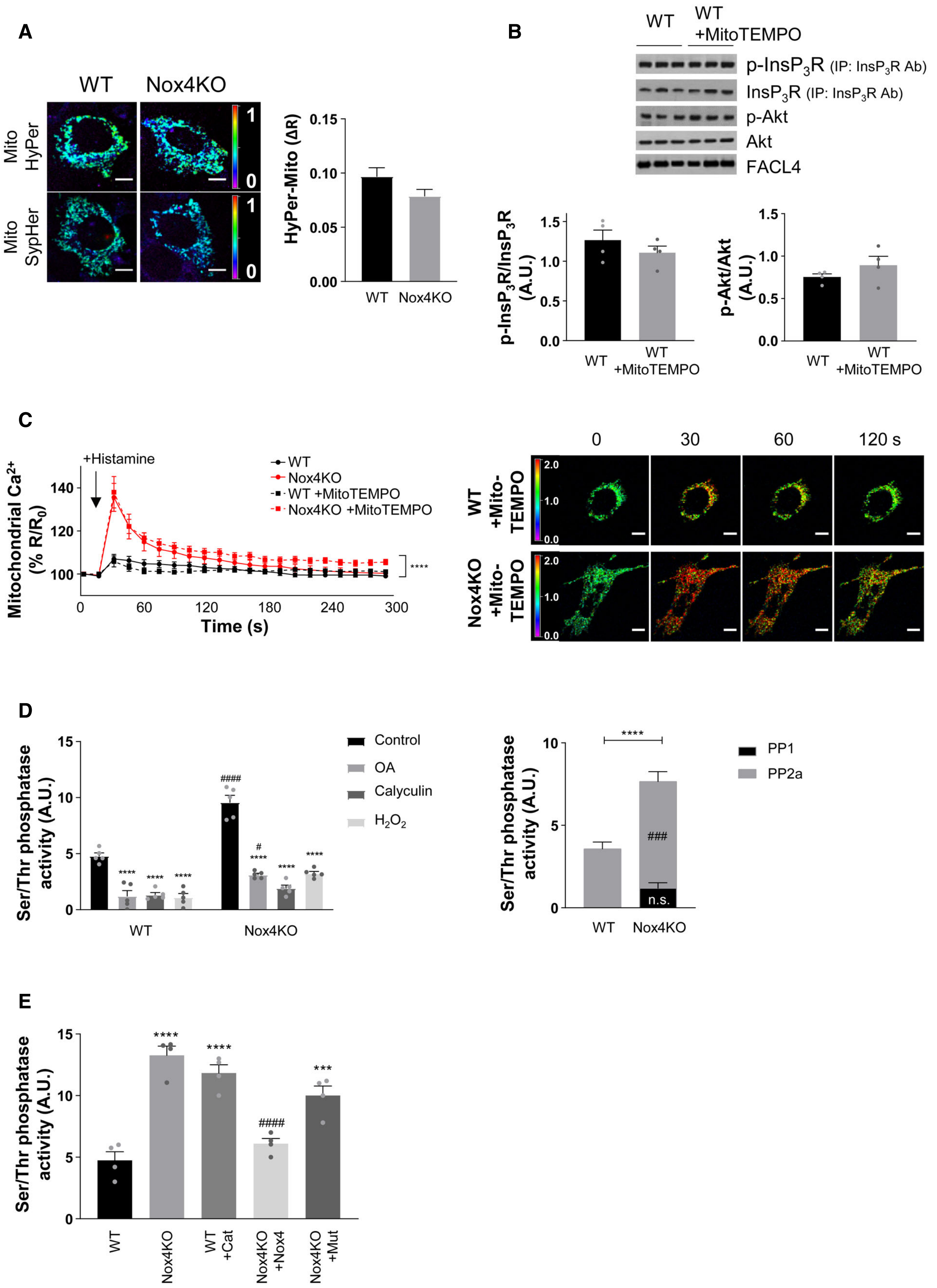

Figure EV4.

 © 2020 The Authors

Figure EV5. Functional effects of Nox4-dependent regulation of $InsP_3R$.

- A Changes in histamine-induced mitochondrial calcium levels in serum-starved WT and Nox4KO MEFs in the presence or absence of Akti or xestospongin C (XeC). Images show the YFP/CFP ratio at different time points. Relates to Fig 6C. Scale bars: 10 μm.
- B Percentage of serum-starved cardiomyocytes with depolarized mitochondria in the absence or presence of XeC. n = 9/group.
- C Cell death in serum-starved cardiomyocytes depleted of Nox4 or control cells, in the absence or presence of XeC. n = 7/group.
- D Changes in histamine-induced mitochondrial calcium levels in serum-starved WT and Nox4KO MEFs in the presence or absence of okadaic acid, as in (A). Scale bars: 10 μm. n = 3/group (with > 50 cells per individual experiment).
- E Cell death in serum-starved WT and Nox4KO MEFs in the presence or absence of okadaic acid. n = 5/group.
- F Indices of cardiac contractile function in perfused WT and Nox4KO hearts at baseline (Basal) and then after 25 min ischemia/30 min reperfusion (recovery). Hearts were treated with XeC (1 μmol/l) or vehicle control for 20 min prior to ischemia. HR = heart rate; dP/dt_{max} and dP/dt_{min} = maximal rate of rise and fall, respectively, of left ventricular (LV) pressure; EDP = LV end-diastolic pressure. n = 6–7/group.

Data information: Data are mean ± SEM. $^{*}P < 0.05$; $^{***}P < 0.001$; $^{****}P < 0.0001$ among compared groups or vs. control. $^{\#}P < 0.05$, $^{\#\#\#\#}P < 0.0001$ vs. Nox4KD or vehicle control. (B,C,E,F): 1-way ANOVA; (D): paired t-test.

© 2020 The Authors

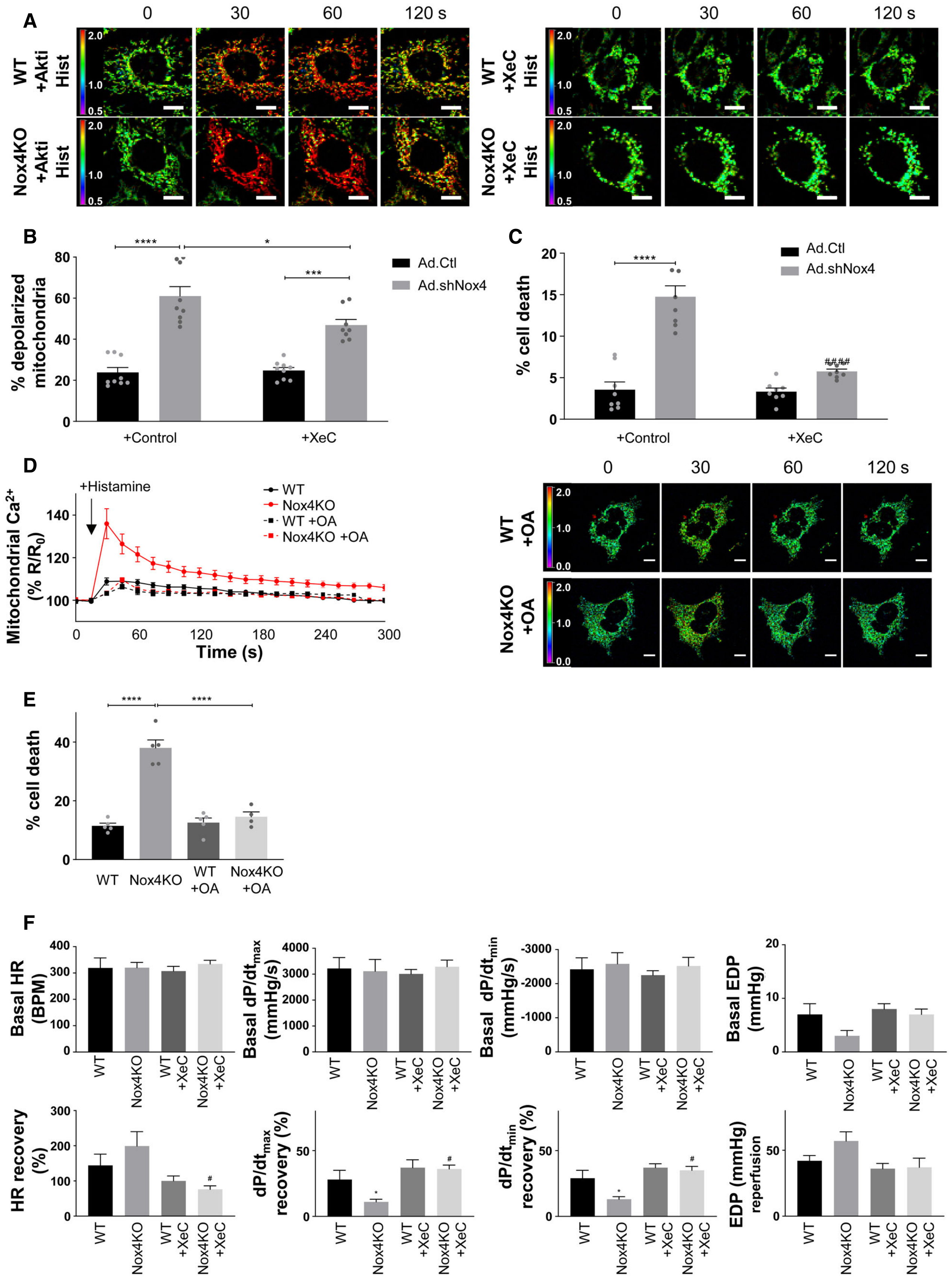

Figure EV5.

© 2020 The Authors
